# Microplastic concentrations, characteristics, and fluxes in water bodies of the Tollense catchment, Germany, with regard to different sampling systems

**DOI:** 10.1007/s11356-021-16106-4

**Published:** 2021-09-17

**Authors:** Matthias Tamminga, Elena Hengstmann, Ann-Kristin Deuke, Elke Kerstin Fischer

**Affiliations:** Center for Earth System Research and Sustainability (CEN), Universität Hamburg, Bundesstraße 55, 20146 Hamburg, Germany

**Keywords:** Freshwater, Lake, Nile red, Data reliability, Pump filtration, micro-Raman-spectroscopy, Mesh size, Cut-off size

## Abstract

The widespread presence of microplastics in multiple environmental compartments has largely been demonstrated. Assessing the ecological risk that microplastics pose is, at the present stage, hindered due to methodical differences. Moreover, different methods hamper meaningful comparisons between studies and data on microplastics <300 μm is scarce. Therefore, we focused on microplastics >20 μm in freshwater and sampling-related aspects in this concern. Sampling was conducted between 2018 and 2020 in the Tollense catchment in northeastern Germany and was carried out by in situ pump filtration. Two different sampling systems (cutoff sizes 20 μm and 63 μm) were applied to filter water volumes of 0.075–1.836 m^3^. Retained particles were analyzed by a combination of Nile red staining and micro-Raman spectroscopy. Thereby, we found microplastic concentrations between 123 and 1728 particles m^−3^ using the 63-μm cut-off size and between 1357 and 2146 particles m^−3^ using the 20-μm cut-off size. Local hydrodynamics (discharge and flow velocity) and land cover are likely influencing the observed microplastic concentrations and fluxes. The variability between both sampling systems cannot fully be explained by the different mesh sizes used. We argue that differentiation between a theoretical cut-off size (finest mesh) and a factual cut-off size (reliable quantification) can help to understand sampling related differences between studies.

**Supplementary Information:**

The online version contains supplementary material available at 10.1007/s11356-021-16106-4.

## Introduction

The widespread presence of microplastics, plastic particles <5 or 1 mm (Arthur et al. 2009; Hartmann et al. 2019), has been documented in various environmental compartments. The affected environmental compartments comprise sediments and soils (Woodall et al. 2014; Vaughan et al. 2017; Corradini et al. 2019), fresh-and seawater (Enders et al. 2015; Faure et al. 2015; Setälä et al. 2016; Kanhai et al. 2018; Bordós et al. 2019; Park et al. 2020), ice (Kanhai et al. 2020), organisms (Leslie et al. 2017; Bessa et al. 2018; Li et al. 2018), and the atmosphere (Cai et al. 2017; Allen et al. 2019; Klein and Fischer 2019).

As the presence of microplastics in the environment is evident, the assessment of associated risks got into focus, recently (GESAMP 2019; de Ruijter et al. 2020; Koelmans et al. 2020). Assessing the risk of a contaminant needs information on its (1) toxicity and (2) environmental abundance (de Ruijter et al. 2020). Moreover, both aspects need to be relatable to one another, for example, by effect thresholds that are linked to environmental concentrations (Koelmans et al. 2020). (1) In terms of toxicity, laboratory-based studies have been carried out for various freshwaters species in the past, but particle sizes, shapes, and polymer types are often simplified and therefore not fully congruent with environmental microplastics (Murphy and Quinn 2018; Redondo-Hasselerharm et al. 2018; Mateos-Cárdenas et al. 2019). To solve this concern, Koelmans et al. (2020) proposed a method for transferring laboratory effect thresholds into environmentally realistic thresholds (in terms of sizes, shapes, and polymer types). (2) Concerning the environmental abundance of microplastics in freshwater, a growing amount of data became available by an increasing number of studies (Eerkes-Medrano and Thompson 2018). However, the majority of these studies relies either on sampling with relatively coarse nets (>300 μm) or, when targeting particles <300 μm, on small sample volumes (Li et al. 2018; Stock et al. 2019; Boyle and Örmeci 2020).

Various studies have shown that microplastic concentrations in water depend on the respective sampling method in general and on the applied mesh size specifically (Vermaire et al. 2017; Cai et al. 2018; Green et al. 2018; Covernton et al. 2019; Tamminga et al. 2019; Lindeque et al. 2020; Prata et al. 2020, 2021a). In this context, Green et al. (2018) compared 1 l grab samples (filter pore size 0.45 μm) to common net sampling techniques (mesh size 200–500 μm), with microplastic concentrations in grab samples being ca. 3 orders of magnitude higher than in net samples. Similarly, Lindeque et al. (2020) found that microplastic concentrations in seawater increased up to 10-fold when comparing nets with mesh sizes of 500 μm and 100 μm for surface trawling. Within these studies, reducing the mesh size was often accompanied by drastically reducing the sample volume. Covernton et al. (2019) identified a decreasing microplastic concentration with increasing sample volume and mesh size by reviewing the available literature. Thereby, both, sampling volume and the applied mesh size, influence reported microplastic concentrations. Moreover, data on microplastics <300 μm based on larger sampling volumes (>10 l) is scarce and methodical differences further limit the comparability of data.

The variability and reliability of reported environmental microplastic concentrations need better comprehension to identify potential risks associated with microplastics. Thus, this study focuses on microplastics smaller than common manta mesh sizes and sampling-related aspects in this concern. We evaluated microplastic concentrations in tributaries and a lake down to a particle size of 20 μm while maintaining a reasonably high sampling volume. Moreover, comparing two sampling systems with different cut-off sizes, we discuss the implications connected to using different mesh sizes on resulting microplastic concentrations and fluxes.

## Material and methods

### Study area and sampling locations

The present study was conducted within the catchment of the river Tollense in northeastern Germany. The Tollense drains a catchment of 1829 km^2^ and is a tributary of the river Peene, which flows into the Baltic Sea (LUNG 2004). The Tollense’s upper catchment is characterized by Lake Tollense that covers 17.9 km^2^ and drains an area of 525 km^2^. Lake Tollense is fed by several small tributaries (mean discharge) of which the Gaetenbach (0.55 m^3^/s), Nonnenbach (0.57 m^3^/s), Liepskanal (0.49 m^3^/s) and Wustrower Bach (0.10 m^3^/s) contribute the largest share of surface inflows (Nixdorf et al. 2004). The only outlet of Lake Tollense is the river Tollense draining the lake at its northern end.

**Fig. 1:**
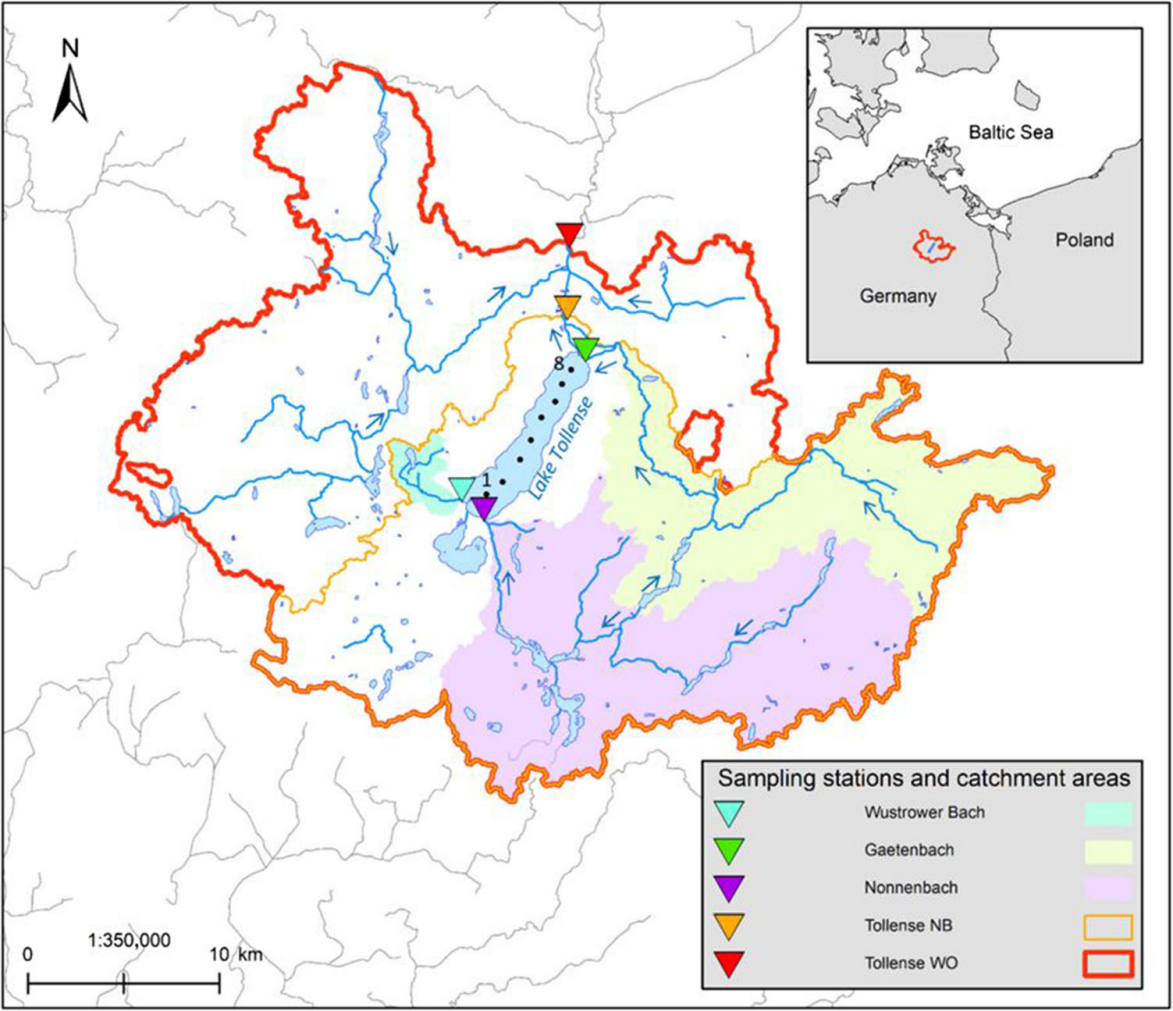
Sub-catchments of the Tollense and its tributaries with respect to sampling locations (triangles). Sampling locations at Lake Tollense are represented by black dots and numbers indicate relative sampling positions from 1 = south to 8 = north. Flow directions are shown as blue arrows. Coordinate system: ETRS 1989 UTM Zone 33N; Water bodies and catchments: LUNG 2021)

A total of five locations were sampled along the course of the Tollense and its tributaries (Fig. 1). Three of these sampling locations are tributaries of Lake Tollense (Gaetenbach, GB; Nonnenbach, NB; and Wustrower Bach, WB), while two sampling locations are situated downstream of the lake (Tollense at Neubrandenburg, TN and Tollense at Woggersin, TW). Additionally, eight locations within Lake Tollense were sampled.

For each sampling location (exclusive lake samples), individual sub-catchments draining toward the respective point were calculated (Fig. 1). Land cover within these sub-catchments was derived from the CORINE land cover data set (EEA 2021; LUNG 2021; see supporting information (SI) 1 for catchment area sizes and SI 2 for land cover distribution within the study area). Agricultural areas and forests dominate land cover within the study area (Table 1). The sub-catchments GB, TN, and TW show higher shares of urban and industrial areas, which can be attributed to the fact that GB, TN, and TW comprise parts of the city of Neubrandenburg. Neubrandenburg has a population of 64,086 and is the economic and touristic center of the area (StatA MV 2019).

**Table 1:** Land cover (%) within the catchment of the Tollense based on individual sampling locations and CORINE land cover data (EEA 2021)

Land cover in %	GB	NB	WB	TN	TW
Agriculture	78.9	72.2	89.7	63.7	69.5
Forest	14.6	22.6	6.6	24.9	20.0
Water	1.1	3.2	3.7	6.0	3.9
Parks and leisure facilities	1.0	0.2	0.0	0.8	0.9
Natural vegetation	0.2	0.6	0.0	1.1	1.4
Industrial or commercial	0.8	0.0	0.0	0.8	1.3
Urban	3.3	1.2	0.0	2.7	3.0

### Sampling

Two different pump filtration systems were used for sampling in this study. The first system (hereinafter 63-μm system) has been used for sampling microplastics in Lake Tollense before and is described in detail in Tamminga et al. (2019). The 63-μm system consists of a submersible pump connected to a cascade of analytical testing sieves (mesh widths: 1.0, 0.63, 0.3, 0.2, and 0.063 mm) via a PVC hose. The pump’s flow rate (verified in the field, cf. Tamminga et al. 2019) was used to quantify the volume of filtered water. The second system (hereinafter 20-μm system) is a commercially available in situ filtration system for sampling microplastics in water (Microplastic particle pump, KC Denmark). The 20-μm system is a modified version of the sampling device described in Karlsson et al. (2020) and Schönlau et al. (2020). It consists of a stainless steel pump placed above a cascade of up to four stainless steel sieves (mesh widths: 0.3, 0.1, 0.05, and 0.02 mm) followed by an inductive flow meter. The 20-μm system is connected to a computer terminal where precise programming of the desired sample volume can be done (see SI 3 for a depiction of the in-field setup).

Sampling has been conducted in March 2018, 2019, and 2020 using the 63-μm system at the Tollense and its tributaries. The sampling station WB could only be sampled in March 2018 due to low water levels in the subsequent years. Moreover, TW was only sampled in March 2019 and 2020. The 20-μm system was used to sample the same stations (except WB) as well as Lake Tollense in September 2019. At Lake Tollense, eight stations distributed across the entire lake (Fig. 1) were sampled (results for the same stations are available from Tamminga et al. 2019 and Tamminga and Fischer 2020).

The infield procedures for both sampling systems were similar. Sampling was carried out at bridges in the case of tributaries and from a small vessel in the case of Lake Tollense. The pumps were lowered toward the water surface using a stainless steel winch (including a stainless steel wire) until the water inlets were fully submerged (0–15 cm below the surface). Applying the 20-μm system, pumping stopped automatically after filtrating 1 m^3^ of water. Concerning the 63-μm system, pumping was stopped manually after eleven minutes (aiming for at least 1 m^3^). At the tributary locations, small particles were sometimes clogging the finest sieves (see supplementary information 4 for sample volumes). If clogging was observed (decrease of the flow rate), pumping was interrupted and sieves were replaced by clean ones (max. twice). If sieves tended to clog again quickly, sampling was continued without the finest sieve. Thereby, using the 63-μm system, between 0.075 and 1.836 m^3^ of water were filtered per station for the smallest size fraction >0.063–0.2 mm. For particles >0.2 mm, between 0.990 and 1.836 m^3^ were filtered with the exception of WB. At WB, the total sample volume did not exceed 0.417 m^3^. The 20-μm system was capable of filtering 1 m^3^ at every station, except NB, where 0.7 m^3^ were filtered for the smallest size fraction >0.02–0.05 mm.

The content of sieves was rinsed into brown glass jars (500 ml) using ultrapure water (finest filter: 0.2 μm). One milliliter of hydrochloric acid (HCl, 37%, VWR) was added to each glass for preservation, and glasses were stored at 4°C until further processing in the laboratory.

At each sampling location, flow velocity and discharge were recorded by an ADC (Acoustic Digital Current Meter, OTT HydroMet GmbH) immediately after the microplastic sample was retrieved.

### Sample purification and QA/QC measures

For digesting biogenic organic matter, the sample material was recovered from brown glass jars and transferred into glass beakers via rinsing with little ultrapure water. Here, we followed a digestion protocol that has been successfully applied in former studies and is described therein in detail (Hengstmann et al. 2018; Tamminga et al. 2018, 2019; see as well SI 5). In brief, the protocol comprises two digestion steps using oxidizing agents at room temperature. First, 60 ml hydrogen peroxide (H_2_O_2_, 30%, Merck) per 50 ml sample volume was added to the beakers. After an exposure time of 7 days, samples were poured through a sieve (mesh width according to the respective sampling system’s finest mesh) to eliminate the remaining H_2_O_2_ and were rinsed into the beakers again. Then, 16.7 ml of sodium hypochlorite (NaClO, 6–14% active chlorine, Merck Emplura) per 50 ml sample volume were added and the reaction was allowed to proceed for 24 h. Finally, samples were filtered onto qualitative filter papers (VWR, qualitative filter paper 413, 5–13 μm particle retention) using a stainless steel filtration funnel. The filters were transferred into glass Petri dishes, covered with a watch glass, and left to dry at room temperature.

Assuring and controlling the quality of analyses is of particular importance in microplastic research (Koelmans et al. 2019). Prata et al. (2021b) analyzed 50 recent publications dealing with microplastics in various environmental compartments regarding ten contamination control parameters. In conclusion, they formulated seven essential aspects of contamination control that were also used as a guideline in this study. Cotton laboratory coats were worn by all personnel; samples were processed in a room with limited access and equipped with an air purifier (Philips, AC3256). Moreover, used materials were made of glass and metal whenever applicable and sieves were washed before as well as in between sample processing, and samples were covered with watch glasses at any time except when directly handled. To account for remaining contamination, procedural laboratory blanks were processed alongside field samples. Blanks underwent the same steps as actual samples, except for starting with 50 ml of ultrapure water. The mean number of microplastics present on blank filters was subtracted from field sample counts.

### Quantification and qualification of microplastics

Microplastics on filters were quantified by Nile red staining. Nile red (Nile red, extra pure, Carl Roth) was solved in chloroform (CHCl_3_, AnalaR NORMAPUR, VWR) as described in Tamminga et al. (2018, 2019). One milliliter of Nile red solution (1 mg/ml) was applied on each filter; filters were covered with a watch glass and dried for at least 24 h at room temperature. Afterwards, filters were photographed under a fluorescence microscope (Zeiss, AxioLab A1, 2.5x/006 A-Plan) connected to a digital camera (Canon EOS 80D, exposure time 1′, ISO 500, 6000 × 4000 μm) and equipped with an external light source (Photonic, F5100Endo, broadband) and a TRITC HC Filter Set (AHF, ex.: 532–554 nm, em.: 573–613). For particle counting, photos were examined in Adobe Photoshop (version CS5) and stained particles were compared to a set of artificial and natural reference materials (see supporting material of Tamminga et al. 2019). Plastics appear in shades of yellow, while natural debris if stained at all, appears in orange to dark red (see Supplementary Information 6 for illustration of the differentiation between microplastics and natural particles as well as assigned Raman spectra). Microplastics were classified as irregular particles (IPs) or fibers according to Hartmann et al. (2019) and their length and width were recorded. Within this study, the sum of both shape categories is referred to using the term particles.

A subset of 336 stained particles (2.24% of all particles) comprising 188 IPs and 148 fibers were subsequently analyzed by μRaman spectroscopy (Thermo Fisher Scientific, DXR2xi Raman Imaging Microscope) to verify their artificial origin and to gain information on their polymeric composition. A 532 nm laser was applied at 5–10 mW using a 25-μm confocal pinhole. Either ×10 or ×50 magnification was used depending on the respective particle size. Spectra were obtained at 10–100 Hz integrating up to 1000 scans. The results were compared to multiple spectral libraries, including artificial and biogenic polymer spectra as well as mineral signals. The best match was chosen for assigning a polymer type to each particle. In general, a match of 70% was perceived as sufficient to accept an assignment, but a trained operator verified the auto-assignments as well.

### Data analysis and processing

All data were analyzed in R (R Core Team 2018) in an RStudio environment (RStudio Team 2018). Plots were generated using the ggplot2 package (Wickham 2016). Geodata were processed in ArcMap (Esri, Version 10.5.1).

Numeric concentrations reported for microplastics in freshwaters often span several orders of magnitude. Besides variability related to actual differences of environmental conditions, methodical discrepancies hamper comparisons between studies. Thus, Koelmans et al. (2020) proposed an alignment method based on the power law character of microplastic size distributions to account for some of the variability between studies targeting different size ranges. Following their proposal, we fitted a linear model to log-log plot of relative microplastic abundances by size classes as described in Kooi and Koelmans (2019). Thereby, we calculated *α*-values of 2.0 (*R*^2^ = 0.914) for the 63-μm system and 2.4 (*R*^2^ = 0.975) for the 20-μm system that were used to determine correction factors in the following.

## Results

Results are described separately for both sampling systems, as distinct differences in measured microplastic concentrations were visible. These differences are discussed in the following.

Blank contamination was deemed acceptable compared to the number of microplastics that were found on filters of field samples. Concerning the 63-μm system, 1.6 ± 2.7 IPs and 1.2 ± 1.5 fibers were detected in blanks on average (±SD, see SI 7 for detailed blank results). For IPs, contamination tended to rise with decreasing size, but for fibers no such trend was visible. Blank samples of the 20-μm system showed a similar size-related distribution. Mean contamination consisted of 10.6 ± 10.0 IPs and 2.1 ± 3.0 fibers. Contamination was higher for the 20-μm system, which is in general expectable due to the increased number of smaller particles (cf. Prata et al. 2021b). Such small particles are also more likely to be transported through the air, thus inducing a higher risk for airborne contamination (e.g., Klein and Fischer 2019). Still, the number of microplastics on filters from field samples exceeded the number of microplastics on blank filters by 8–20 times.

### Results applying the 63-μm system

Microplastic concentrations were in a similar range when comparing the three sampling campaigns in which the 63-μm system was used (Table 2). Microplastic concentration was highest in March 2019, followed by March 2020 and March 2018. Microplastics were dominantly composed of IPs (mean ± SD share: 80 ± 11% IPs and 20 ± 11% fibers). Moreover, microplastic concentrations were more variable in March 2019 when compared to March 2020 and March 2018. This was mainly due to the concentration measured at GB in March 2019, which was considerably higher compared to the other sampling points.

**Table 2:** Microplastic concentrations by sampling device, sampling campaign, and particle shape (*Fib*. fibers; *Par*. particles) expressed in particles per m^*3*^

	63-μm system						20-μm system					
	March 2018			March 2019			March 2020			September 2019		
	IPs	Fib.	Par.	IPs	Fib.	Par.	IPs	Fib.	Par.	IPs	Fib.	Par.
Mean	247	39	**286**	512	56	**567**	403	85	**488**	1612	200	**1812**
Median	183	37	**224**	160	49	**209**	228	85	**349**	1620	195	**1872**
SD	175	18	**176**	752	29	**778**	398	45	**388**	333	60	**339**

**Fig. 2:**
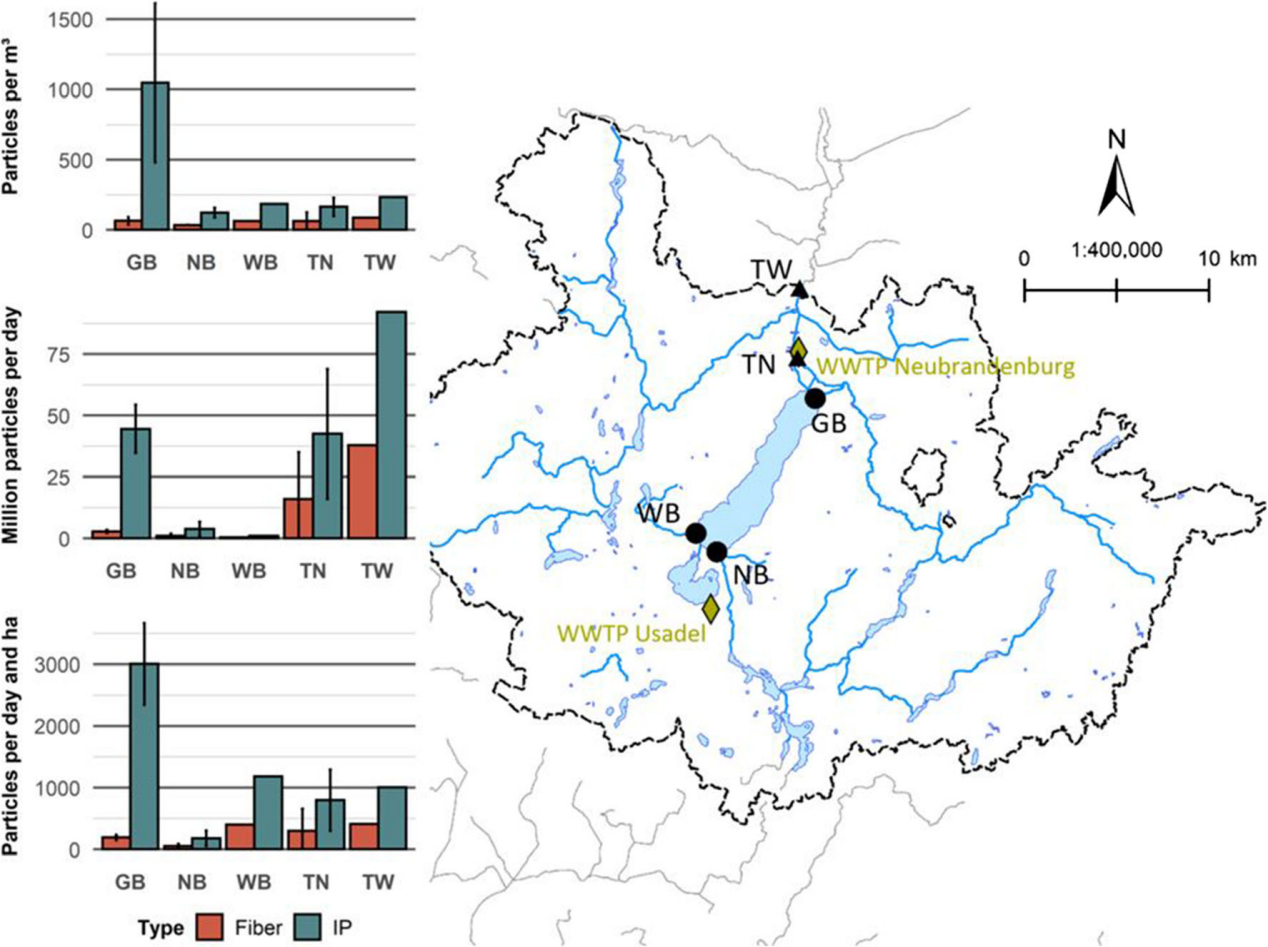
Spatial distribution of microplastics expressed as particle concentrations and fluxes by shape categories and sampling location. Error bars indicate the standard deviation. Sampling locations are displayed as upstream (circles) or downstream (triangles) of Lake Tollense. Yellow diamonds represent wastewater treatment plants (WWTP). Note that the WWTP in Neubrandenburg is the only larger WWTP in the area with a population equivalent (p.e.) of 100,000 inhabitants (WWTP Usadel p.e.: 1400 inhabitants). Coordinate System: ETRS 1989 UTM Zone 33N; water bodies and catchments: LUNG 2021)

Figure 2 displays the spatial distribution of microplastics retrieved with the 63-μm system. Microplastic concentrations at GB were by far above those at other sampling locations. Moreover, a slight increase of microplastic concentrations toward downstream locations within the catchment is visible. Mean (median where applicable) concentrations were 1110 (1059) particles m^−3^ at GB, 320 particles m^−3^ at TW, 247 particles m^−3^ at WB (single measurement), 225 (200) particles m^−3^ at TN, and 157 (154) particles m^−3^ at NB. GB showed the highest microplastic concentrations across all sampling campaigns, while NB showed the lowest concentrations in March 2019 and 2020. In March 2019, the lowest concentration was measured at TN (123 particles m^−3^), but the concentration at NB was only slightly higher (124 particles m^−3^).

Based on the discharge measured in the field (available in SI 8), we calculated microplastic fluxes by multiplying concentrations (particles m^−3^) with discharge values (m^3^/s). Subsequently, results were referred to a flow of particles per day. The derived fluxes differed considerably across sampling locations and tended to increase toward downstream locations within the catchment. Mean (median where applicable) fluxes were 129.9 million particles per day at TW, 58.4 (58.5) million particles per day at TN, 47.4 (42.6) million particles per day at GB, 5.0 (5.3) million particles per day at NB, and 1.5 million particles per day at WB. TW showed the highest microplastic fluxes in all sampling campaigns (not sampled in March 2018), while the lowest fluxes were calculated for NB (excluding WB).

Calculating absolute flux values enables generating a simple budget for in- and outputs of microplastics at Lake Tollense. TN is located close to the only outlet of Lake Tollense and can therefore be used to approximate outputs of microplastics from the lake. All major tributaries of Lake Tollense were sampled as well (GB, NB, and WB). The balance was then calculated by subtracting the output fluxes from the sum of the input fluxes. In March 2018 (Δinputs-output: −6.0 million particles per day) and 2020 (−35.5 million particles per day), microplastic fluxes out of Lake Tollense exceeded inputs. In contrast, input fluxes were higher than the output flux in March 2019 (24.8 million particles per day).

Flux values were further normed to the catchment size (hectare) to allow for evaluating the pollution intensity with respect to land cover patterns. The ranking of the sampling locations changes when considering particle fluxes per day and hectare. In this manner, GB is characterized by the highest flux rates, followed by WB, TW, TN, and NB. With a mean of 227 particles per day and hectare, NB is well below fluxes at the other locations (>1000 particles per day and hectare).

**Fig. 3:**
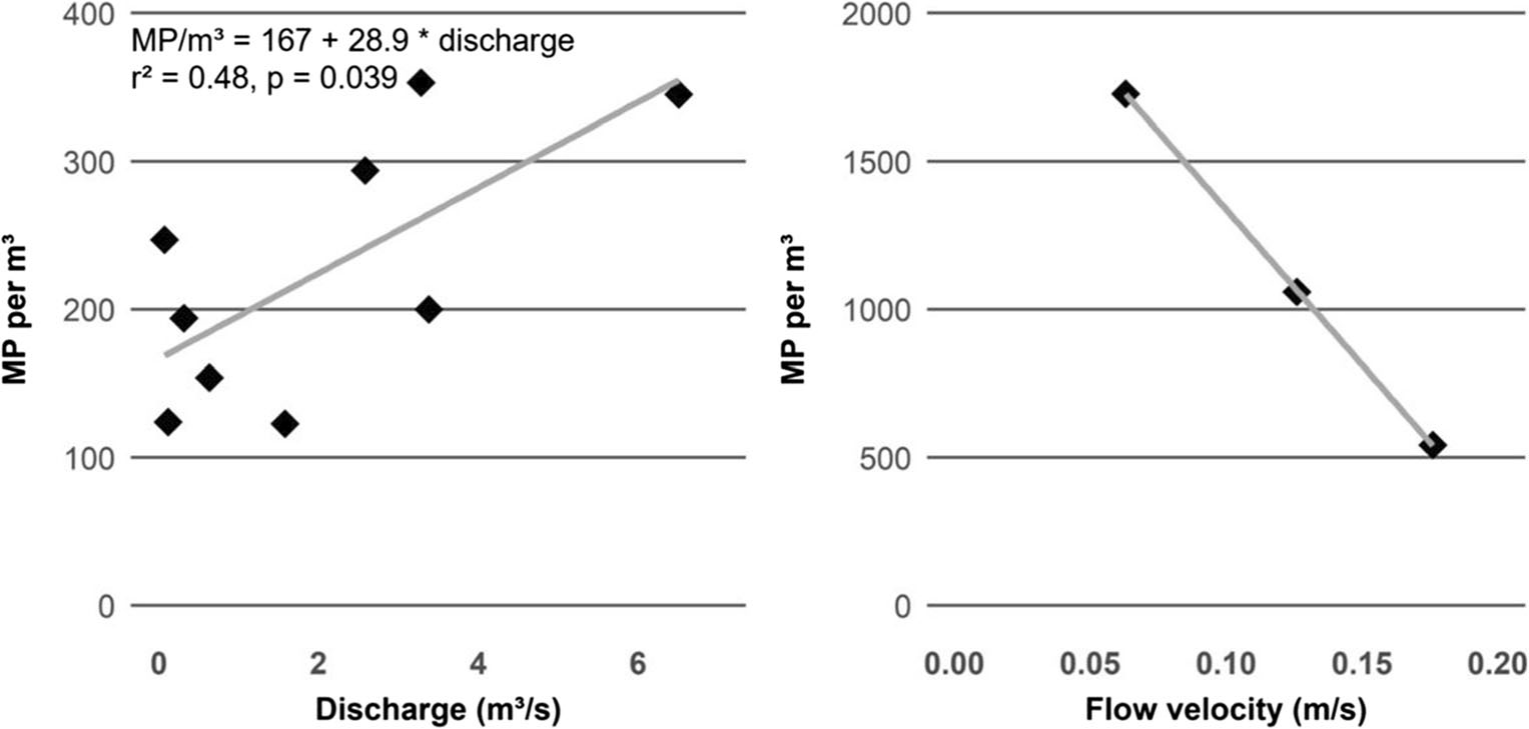
Microplastic concentrations in relation to the current discharge at sampling locations for all locations except GB (left) and microplastic concentrations in relation to the mean flow velocity at GB (right)

Microplastic concentrations showed a significant (*p* < 0.05) positive correlation (*r* = 0.69) with discharge data (Fig. 3, left). This correlation was valid at all sampling locations except for GB. Here, microplastic concentrations tended to decline with rising discharge. This tendency was more pronounced when choosing the mean flow velocity instead of discharge (Fig. 3, right).

### Results applying the 20-μm system

Microplastic concentrations retrieved with the 20-μm system were considerably above values of the 63-μm system (Tab. 2). Again, microplastics were dominantly composed of IPs (mean ± SD share: 89 ± 3% IPs and 13 ± 4% fibers), but the share of fibers was even lower than using the 63-μm system. The spatial distribution was similar to the March sampling campaigns when the 63-μm system was applied. The highest concentration was measured at GB (2146 particles m^−3^) and the lowest concentration was measured at TW (1357 particles m^−3^).

In principle, the spatial distribution of microplastic fluxes retrieved with the 20-μm system was similar to patterns observed with the 63-μm system. The highest fluxes were observed at the downstream locations TN (120.1 million particles per day) and TW (109.7 million particles per day). Concerning the tributaries of Lake Tollense, fluxes at GB (15.6 million particles per day) were above those at NB (2.0 million particles per day). With −103.2 million particles per day, the deficit of the input-output balance is more pronounced compared to values gained with the 63-μm system. Fluxes normed to catchments were higher at the downstream locations TN (2267 particles per day and hectare) and TW (1195 particles per day and hectare) when compared to GB (1050 particles per day and hectare) and NB (91 particles per day and hectare).

Within Lake Tollense, microplastic concentrations were three- to fourfold lower than in its tributaries and the Tollense. The mean (median) concentration was 489 (496) particles m^−3^. No distinct spatial pattern of microplastic concentrations in the lake was observed (see supplementary information 9). The highest concentration was measured at Station L7 (625 particles m^−3^) in the north of the lake, while station L4 in the center of Lake Tollense showed the lowest microplastic abundance (234 particles m^−3^).

### Polymeric composition and particle size distribution

**Fig. 4:**
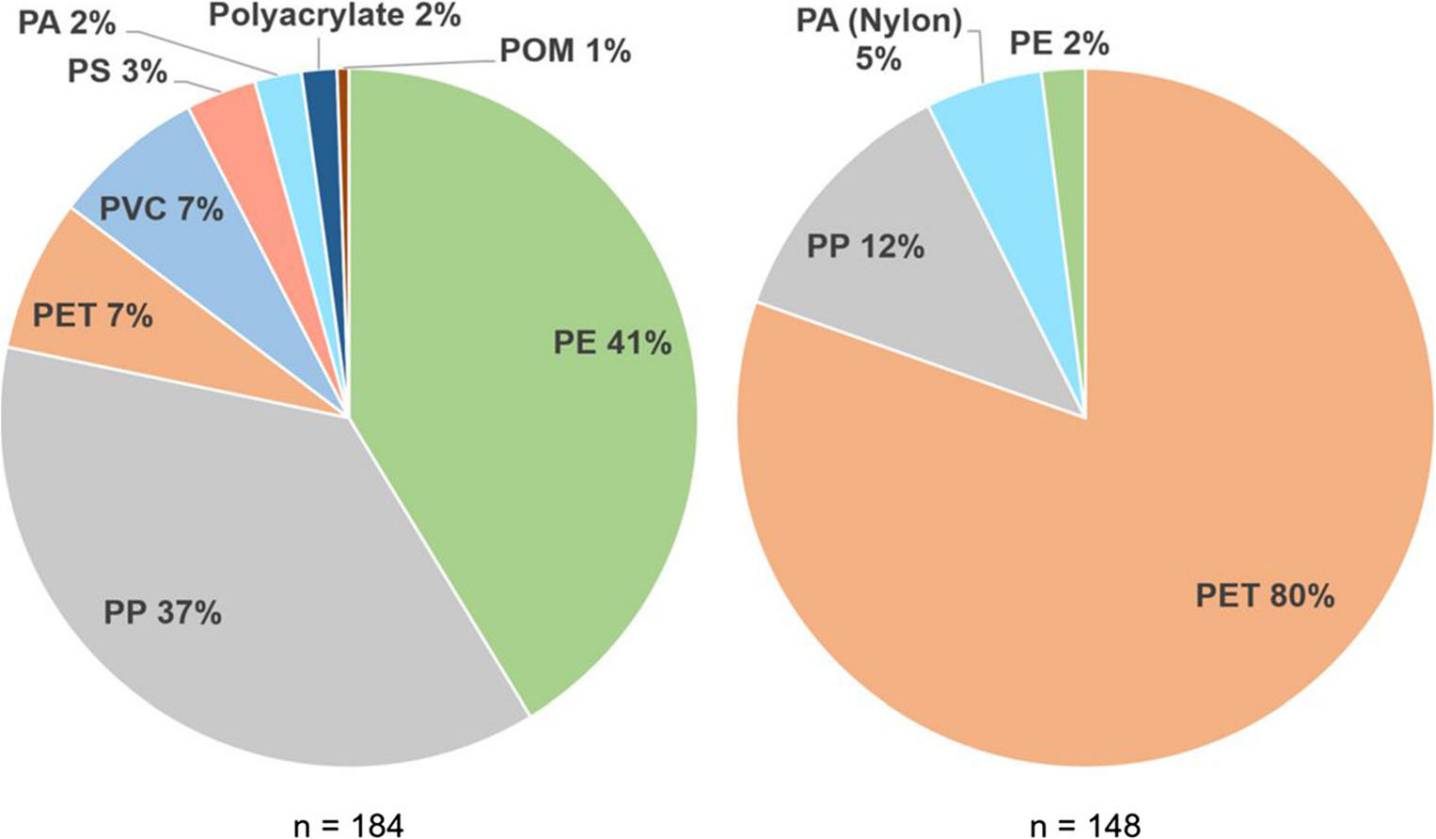
Polymeric composition of particles from the Tollense catchment for IPs (left) and fibers (right)

The results of the spectroscopic analysis were summarized for both sampling systems to enlarge the sample size (Fig. 4). Of the analyzed particles, 332 were verified as artificial polymers, three did not show a conclusive signal and one particle was composed of cellulose. Mean (SD) match quality was 84 ± 12% and the mean signal-to-noise ratio was 69 ± 44.

Particle shape did significantly influence the material composition. IPs were dominantly composed of polyolefins (78%) and to a lesser degree of PET (polyethylene terephthalate) and PVC (polyvinyl chloride). PS (polystyrene) was only observed in samples of TW in the form of spherical particles. The chemical composition of fibers was less variable with the majority of fibers being made of polyester. PP (polypropylene) fibers had considerably larger diameters (>40 μm) compared to PET fibers (mostly below 30-μm in diameter).

Particle size distributions by sampling campaign and particle shape are displayed in the supporting information (SI 10). In general, size distributions differed according to the particle shape. IP abundance was exponentially increasing with decreasing particle size, except for particles whose size was close to the mesh size of the respective sampling system. In contrast, fibers were widespread across the size range and showed the highest abundance between 300 and 500 μm.

Particle size distributions were similar for both sampling systems concerning their principal structure. As expected, particles were larger in samples of the 63-μm system. Mean ± SD fiber length was 1007 ± 700 μm for the 63-μm system and 727 ± 673 μm for the 20-μm system (fiber diameters: 19 ± 11 μm and 17 ± 9 μm). Likewise, the average IP was 229 ± 217 μm long and 106 ± 77 wide within samples of the 63-μm system, compared to 104 ± 101 μm and 47 ± 35 μm for the 20-μm system.

## Discussion

### Comparison with other studies

Numerical concentrations for microplastics in freshwaters differ vastly among studies, spanning several orders of magnitude on a global scale (Cera et al. 2020; Scherer et al. 2020). To compare data of this study with previous findings, a selection of studies was chosen based on either methodical and/or regional comparability (Table 3). Moreover, a correction following Koelmans et al. (2020) was applied to compensate for a share of the variability induced by different size ranges targeted. Applying this correction, microplastic concentrations measured by both the 63-μm system and the 20-μm system are in a comparable range. Due to this conformity, the general comparison to previous studies is carried out jointly for the two sampling systems. The comparison is further conducted with respect to the water body type, which means that microplastic concentrations of Lake Tollense are compared to studies carried out in lakes, only. Results from tributaries of the Tollense catchment were analogously compared to studies carried out in rivers.

**Table 3:** Comparison of microplastic abundance in freshwaters. Temperature (temp.) refers to the highest temperature applied. For comparison with other studies values at GB were excluded (see below). *Mean of sampled volumes, **original values were reported in MP/L, ***following Koelmans et al. 2020

Reference	Country	Type	Sampling			Sample processing		Conc. (MP/m^3^)	This study (corrected)***	
			Sampling method	Sample volume (l)	Cut-off (μm)	Digestion/temp.	Identification	Mean/median min–max	63 μm-system (min–max)	20 μm-system (min–max)
Mao et al. 2020	CHN	River	Pump, filtration	50	64	H_2_O_2_ (30%)/100 °C	Nile red, Raman	n.r. 7–700**	121–347	272–430
Pan et al. 2020	CHN	River	Metal bucket, filtration	20	330	H_2_O_2_ (30%) with 0.05 M FeSO_4_	Visual, Raman	246/n.r. 50–275	22–64	28–44
Stanton et al. 2020	GBR	River	Metal bucket, filtration	30	63	H_2_O_2_ (30%)	Visual, FTIR	n.r. 0–400**	-	278–440
Scherer et al. 2020	GER	River	Plankton net	3200–32,700	150	KOH (10 M) and H_2_O_2_ (30%)	>500 μm: visual, FTIR; >150 μm: visual, FTIR	5.57/5.11 0.9–13.24	51–146	83–132
Heß et al. 2018	GER	River	Manta-Trawl	n.r.	300	<500 μm: enzymes	>500 μm: visual, FTIR <500 μm: FTIR	37.8/19.2 2.9–214.2	25–71	32–50
Eo et al. 2019	KOR	River	Beaker, Pump	100	20	H_2_O_2_ (35%) with 0.05 M Fe(II)/~75 °C	Visual, FTIR	n.r. 293–4760	391–1121	1357–2146
Park et al. 2020										
	KOR	River	Pump, filtration	100	100	H_2_O_2_ (30%)/62.5 °C	FTIR	n.r. 0–234.5	77–221	147–232
Mintenig et al. 2020	NLD	River	Pump, filtration	>100 μm: 1300–8000; >20 μm: 30–2250	20	SDS (5%), KOH (10%), H_2_O_2_ (32%)/35 °C	>300 μm: visual, FTIR; <300 μm: FTIR	n.r./862 67–11,532	391–1121	1357–2146
Sekudewicz et al. 2021	POL	River	Metal bucket, filtration	20	55	H_2_O_2_ (30%)	Visual, Raman/FTIR	n.r. 1600–2550**	141–405	336–532
Rodrigues et al. 2018	PRT	River	Pump, filtration	~1200	55	H_2_O_2_ (30%) with 0.05 M Fe(II)/~75 °C	Visual, FTIR	n.r. 58–1265	141–405	336–532
*This study*	GER	River	Pump, filtration	>200 μm: 990–1836; >63 μm: 75–1836	63	H2O_2_ (30%) and NaClO (6–14%)	Nile red, Raman	226/200 123–353	-	278–440
*This study*	GER	River	Pump, filtration	>50 μm: 1000; >20 μm: 700–1000	20	H_2_O_2_ (30%) and NaClO (6–14%)	Nile red, Raman	1812/1872 1357–2146	391–1121	-
Uurasjärvi et al. 2019	FIN	Lake	Pump, filtration	6–468*	20	n.a.	Visual, FTIR	168.8/n.r. n.r.	-	-
LfU 2019	GER	Lake	Manta trawl	n.r.	300	<500/1000 μm: enzymes	>500/1,000 μm: visual, FTIR <500/1000 μm: SWIR, FTIR	n.r./4 <1–42	-	5–15
Bordos et al. 2018	HUN	Lake River	Pump, filtration	1012–1990	100	H_2_O_2_ (30%)/80 °C	Visual, FTIR	13.8/n.r. 3.5–32.1	77-221	Lake: 25–68 river: 147–232
*This study*	GER	Lake	Pump, filtration	1000	20	H_2_O_2_ (30%) and NaClO (6–14%)	Nile red, Raman	489/496 234–625	-	-

Microplastic concentrations in the Tollense and its tributaries are in an equal range as in earlier studies. Most of the studies reporting similar results used a similar methodical setup concerning the cut-off size of the sampling device (Rodrigues et al. 2018; Eo et al. 2019; Mintenig et al. 2020; Park et al. 2020; Stanton et al. 2020). Mintenig et al. (2020) found concentrations between 67 and 11,532 particles m^−3^ investigating microplastics in the Dommel and Meuse (the Netherlands). These comparably high numbers are similar to our results. This can be explained by the fact that both studies used lower size limits down to 20-μm while maintaining a reasonably high sample volume. Likewise, Rodrigues et al. (2018) found microplastic concentrations of up to 1265 particles m^−3^ in the Portuguese Antuã River using a 55-μm mesh and sampling more than 1 m^3^ of water. Finally, Eo et al. (2019) reported microplastic concentrations between 293 and 4760 particles m^−3^ in the Korean Nakdong River, filtering 100 l of river water through a 20-μm mesh.

Methodological differences seem to account for an important part of the variability between studies, as differences mainly occur when applied methods differ. Among those studies reporting lower concentrations than this study, some applied a visual preselection before particles were spectroscopically qualified (Mani et al. 2015; Mao et al. 2020; Scherer et al. 2020). This may lead to underestimating particles that are transparent, colored similarly to organic debris, or bleached due to aging by UV irradiation. Moreover, in some cases, comparably intensive digestion methods (in terms of applied chemicals or temperatures) were implemented (Baldwin et al. 2016; Scherer et al. 2020). The degradation of plastics in this process may cause an underestimation of the actual microplastics abundance (cf. Munno et al. 2018; Pfeiffer and Fischer 2020). Mao et al. (2020), for example, sampled various stations along the Yulin River, a tributary of the Yangtze River in China, applying similar methods as this study in terms of sampling (pump filtration), quantification (Nile red), and qualification (μRaman). Considering that the authors state to expect high abundances of microplastics due to the insufficient waste management in their study area, it is rather surprising that the corrected values of this study are in a similar range. Still, high temperatures (100 °C) were applied for digesting organic matter in the samples from the Yulin River so that a degradation of polymers is plausible. This is strengthened by the fact that only a few polymer types (PE, PP, and PS) were positively identified. Studies characterized by comparably low sample volumes report higher microplastic concentrations than this study (Pan et al. 2020; Sekudewicz et al. 2021). Extrapolating results based on few liters to one cubic meter may lead to overestimating the microplastic abundance due to a non-representative sample volume.

Microplastic concentrations in Lake Tollense are in a comparable range as previous reports for Bavarian Lakes (LfU 2019). However, reported concentrations for the Bavarian lakes included microplastics below the sampling-related cut-off size (300-μm) and may thus be not fully comparable to values from Lake Tollense. Uurasjärvi et al. (2019) sampled microplastics in Lake Kallavesi, Finland, using a similar in situ pump filtration approach (cut-off size 20-μm) as this study. Microplastic concentrations in Lake Kallavesi are below those for Lake Tollense. Still, the authors state that their actual cut-off size might be higher than 20-μm due to the applied optical microscopy and the manual selection of particles. Thereby, the actual microplastic concentrations in Lake Kallavesi might be higher than reported.

Numerical fluxes of microplastics in freshwater are rarely stated. Among the considered studies, only Stanton et al. (2020) and Eo et al. (2019) gave information on particle flows. Stanton et al. (2020) reported fluxes between 0 and 463 million particles per day for the rivers Leen, Soar, and Trent (UK). The overall range is thereby comparable to fluxes for the Tollense and its tributaries. While zero fluxes might be due to the low sample volume (30 l) enhancing the probability of not collecting any microplastics, maximum fluxes are roughly 3–4 times higher than in the Tollense (at TW). This coincides with the difference in the discharge between TW (mean: 4.5 m^3^/s) and the river Soar (11.7 m^3^/s), where the maximum fluxes were measured. Eo et al. (2019) reported annual fluxes of 1.7 × 10^13^ particles for the Nakdong River in South Korea based on surface sampling and covering four different seasons. This would roughly correspond to 47 billion particles per day, assuming equal fluxes throughout the year. As Eo et al. (2019) found a considerable seasonal variability, this comparison must be interpreted with caution. Still, fluxes within the Nakdong River are at least two orders of magnitude higher than the maximum fluxes observed within the Tollense catchment. This difference coincides with the differences in catchment size (Nakdong River: 21.588 km^2^, Tollense at TW: 918 km^2^). Additionally, the Nakdong River catchment is densely populated containing major cities like Daegu and Busan (Eo et al. 2019).

### Comparison of tributaries and potential sources

Concentrations and fluxes within the Tollense catchment are spatially variable. The sampling station GB showed exceptionally high pollution levels, which cannot be explained by the sole size of its catchment area. These high pollution levels are plausible due to different reasons: (1) the highest share of urban area among all investigated catchments (see Table 1) characterizes the catchment of the GB. Moreover, the last stretch of the GB is located in Neubrandenburg, the economic and touristic center of the region. Several studies have emphasized a relation of urbanization toward pollution levels (Mani et al. 2015; Baldwin et al. 2016; Kataoka et al. 2019). (2) The construction of a road bridge crossing the GB started in early 2018, approximately 2 km upstream of the sampling location. This construction site may have acted as a continuous point source for microplastics in the GB by ongoing activity on-site until the end of 2019. Construction sites are considered an important source for microplastics due to the processing of plastic materials (e.g., insulation and pipes) or the abrasion of plastics from machinery and tools (Bertling et al. 2018). (3) The hydrodynamic conditions in the lower part of the GB may promote the accumulation of microplastics. Measured discharges and especially the mean flow velocities (0.06–0.18 m/s) were comparably low at GB (see SI 8). In contrast to all other sampling locations, lower flow velocities coincide with higher microplastic concentrations at GB (see Fig. 3). Winds are typically blowing from southwesterly directions in the study area, which was, in fact, the case at days before sampling (see SI 11). The last trench of the GB faces southwest so that water from Lake Tollense can be pushed into the GB. Hence, microplastics might be enriched here. This is strengthened by the fact that occasionally surficial upstream flows in opposite directions were observed in the field at GB.

At all sampling locations except GB, a positive correlation of microplastic concentrations with the current discharge was observed, which is plausible since dynamic hydrological conditions lead to the increased mobilization of microplastics within the riparian zone of receiving waters. Discharge within the Tollense catchment is generally controlled by precipitation. It was demonstrated before that rainfall can lead to increased microplastic concentrations within freshwaters (Hitchcock 2020; Xia et al. 2020).

The relation of microplastic concentrations to land cover patterns within the sub-catchments is difficult, while fluxes show a better concordance with land cover. Urban areas, as centers for human activities, concentrate sources for microplastic emissions (Mani et al. 2015; Baldwin et al. 2016; Kataoka et al. 2019). Accordingly, the area (in hectares) covered by the urban land cover class coincides with the mean absolute fluxes (million particles per day) within the sampled catchments. Moreover, mean fluxes normed to the catchment’s size coincide with the share of urban land cover within the respective catchment (with the exception of WB).

Among the polymers found in samples of the Tollense catchment PE and PP dominate beyond their market demand concerning IPs (cf. PlasticsEurope 2020). Both polymers are widely used in various applications but are also strongly connected to consumer-related applications such as food wrapping/packaging and single-use items (Jones et al. 2020; PlasticsEurope 2020). These consumer-related applications are plausible sources of microplastics within the Tollense catchment due to the absence of large-scale industry. Moreover, land cover within the study area is dominated by agriculture, and PE and PP are widely used in related applications (e.g., plastic foils for silage bales). Among all samples, 13 particles were identified as PVC. Two of those particles originated from samples of the 63-μm system. Consequently, it cannot be ruled out that these particles originate from the used PVC-hose. The majority of analyzed fibers were composed of polyester, which is largely applied for clothing. PP fibers, having the second-highest share, were related to shipping and fishing before (Song et al. 2018). Both activities are carried out within the Tollense catchment (especially on Lake Tollense) but at low intensities.

### Comparison of sampling devices and methodical considerations

Microplastic concentrations were severely different when comparing the two sampling systems with respect to the lower cut-off size. It has been emphasized before that lowering the mesh size of a sampling system may lead to significantly higher microplastic abundances (e.g., Dris et al. 2018; Green et al. 2018; Covernton et al. 2019; Prata et al. 2020, 2021a). However, we were able to achieve comparably high sampling volumes for both systems, despite lowering the mesh size in this study. Comparing the mean concentrations of microplastics derived by the 63-μm system to concentrations of the 20-μm system, values are 1.9–11.3 (mean 6.5) times higher in samples of the 20-μm system. Using the alignment method suggested by Koelmans et al. (2020) with an *α* of 2.4 concentrations 5 times higher would be expected using a 20-μm instead of 63-μm mesh. An important share of the discrepancy is therefore related to the targeted size range. Still, taking into consideration that the correction factor would be smaller using the α-value of the 63-μm system (2.0), less difference would be explained. Thus, extrapolating microplastic abundance toward smaller size fractions may underestimate the actual abundance.

When comparing both systems, a noticeable difference became apparent concerning the input-output balance of Lake Tollense, which was considerably higher applying the 20-μm system. Seasonal patterns may influence such results, as for the comparison of the sampling systems in general, but based on present data, seasonal differences cannot be assessed. We rather hypothesize that the larger input-output difference is related to atmospheric inputs on Lake Tollense and those parts of its catchment that were not included by sampling its tributaries. Klein and Fischer (2019) showed that some hundreds of particles per m^2^ and day might be deposited through the atmosphere (both dry and wet deposition) and that the majority of these particles were <63 μm in size. The general dimension of these deposition rates is comparable to other studies (Cai et al. 2017; Allen et al. 2019). Thereby, billions rather than millions of particles may be deposited considering the surface of Lake Tollense alone (17.9 km^2^). While the exact numbers are somewhat speculative, this points to the fact that the importance of input pathways may considerably shift when very small particles are targeted.

For both systems, we observed that particles with a size (length) close to the mesh width of the finest sieve were less effectively collected compared to larger particles, which is in line with previous studies (Heß et al. 2018; Stanton et al. 2020). Particles close to the cut-off size of a system cannot be reliably quantified and thus, a difference between a theoretical and a factual cut-off size exists. While the first refers to the minimum mesh opening of a system, the latter is the size limit at which particles larger than this limit can be reliably quantified. The factual cut-off size can be approximated using the width of a particle, as it will better explain whether a particle may pass a sieve compared to using length (for particles may align in flow direction). In this study, we calculated a mean width-to-length ratio of 0.46 for the 63-μm system and 0.45 for the 20-μm system. These values are comparable to previous studies (0.67 in Simon et al. 2018 and 0.56 in Mintenig et al. 2020). Conversely, this implies a factual cut-off size that may be up to twice as large as the mesh width of the finest size (the theoretical cut-off size). This assumption may not be applicable to spherical particles, as these have higher width-to-length ratios. Moreover, especially long fibers can be collected using mesh widths far above their diameter and it is rather their length (often several 100-μm) that increases the probability that they are retained.

## Conclusions

This study analyzed the abundance, characteristics, and fluxes of microplastics within the Tollense catchment using two different sampling systems. Microplastic concentrations ranged between 123 and 1728 particles m^−3^ using the 63-μm system and between 1357 and 2146 particles m^−3^ using the 20-μm system. Microplastic abundance significantly increased with rising discharge in the tributaries of the catchment. Moreover, higher microplastic concentrations were found in those tributaries that had a higher proportion of urban land cover.

Microplastic concentrations showed a large variability when comparing the two sampling systems used in this study. Sampling with the 20-μm system yielded in up to 10 times higher microplastic concentrations than sampling with 63-μm system. This variability can mostly be explained by the different cut-off sizes being used. Still, the applied correction, precisely its *α*-value, is influenced by the respective mesh size (higher *α*-value for smaller mesh size). Therefore, extrapolating the microplastic abundance toward smaller size fractions may underestimate the actual abundance. Considering the particle size distributions of both sampling systems, we suggest differentiating between a theoretical cut-off size (finest mesh applied) and a factual cut-off size (size limit for reliable quantification) in future studies. More research is needed to verify if the observed difference is site-specific or can be generalized. Consequently, whether environmental microplastic concentrations exceed effect thresholds or not can be related to sampling methods and the assessment of ecological risks may change when sampling is adapted to smaller microplastics.

## Supplementary Information

ESM 1 (DOCX 1844 kb)
