# Supplementary material for: Microplastic concentrations, characteristics, and fluxes in water bodies of the Tollense catchment, Germany, with regard to different sampling systems
Source: Environ Sci Pollut Res Int. 2021 Sep 17;29(8):11345–58. doi: 10.1007/s11356-021-16106-4 (PMC8794927; doi:10.1007/s11356-021-16106-4)
Supplement: Supplementary file 1 — (DOCX 1844 kb) [file 11356_2021_16106_MOESM1_ESM.docx]

**Supplementary to Tamminga et al. 2021: Microplastic concentrations, characteristics, and fluxes in water bodies of the Tollense catchment, Germany, with regard to different sampling systems**

| **Catchment** | **Area (km²)** | **Area (ha)** |
| --- | --- | --- |
| **Wustrower Bach** | 9.33 | 933 |
| **Nonnenbach** | 218.97 | 21897 |
| **Gaetenbach** | 148.28 | 14828 |
| **Tollense at Neubrandenburg** | 532.7 | 53270 |
| **Tollense at Woggersin** | 917.7 | 91770 |

**SI 1** Size of investigated catchments


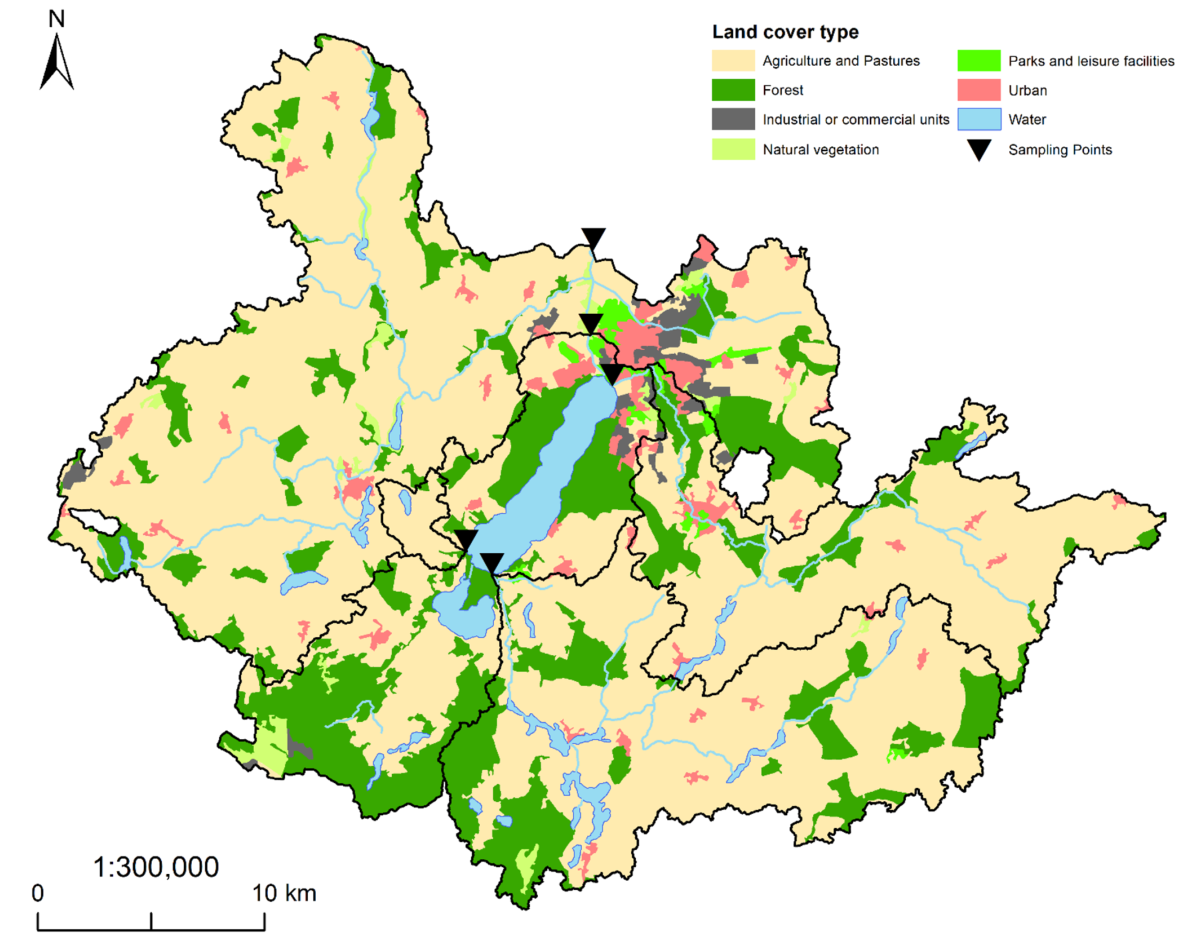


**SI 2** Landcover within the study area based on the CORINE land cover data set (EEA 2021, LUNG 2021)


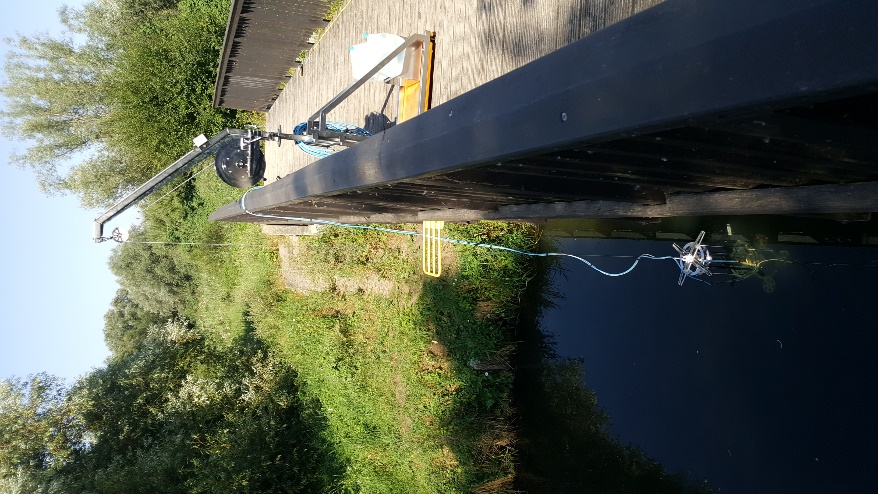


**SI 3** Set-up of the 20 µm-system in the field

| **Sampling station** | **Mar. 2018** | **Mar. 2019** | **Sep. 2019*** | **Mar. 2020** |
| --- | --- | --- | --- | --- |
| Gaetenbach | 990 (97) | 1069 (75) | 1000 | 1163 (183) |
| Nonnenbach | 1066 (321) | 1043 | 1000 (700) | 1152 |
| Wustrower Bach | 417 | - | - | - |
| Tollense NB | 1027 (786) | 1836 | 1000 | 1228 |
| Tollense WO | - | 1305 | 1000 | 1260 |
| Lake Tollense | - | - | 1000 | - |

**SI 4** Sample volumes (in liter) by sampling stations and sample time, numbers in brackets give sample volumes for the finest sieves, if clogging hindered the filtrations of 1,000 l,
*20 µm-system

**SI 5** Digestion protocol applied in this study according to Hengstmann et al. 2018 and Tamminga et al. 2018, 2019

| For digesting biogenic organic matter, the sample material was recovered from brown glass jars and transferred into glass beakers via rinsing with little ultrapure water. At first, hydrogen peroxide (H⁠_2_O⁠_2_, 30%, Merck, 60 ml per 50 ml sample volume) was added to the sample. The beaker was covered with a watch glass and incubated for seven days at room temperature. Subsequently, the hydrogen peroxide was removed by pouring the beaker content through an analytical sieve (63 µm or 20 µm) and rinsing with little ultrapure water. After transferring the sample into the beaker again by rinsing with little ultrapure water, the sample was treated with 16.7 ml sodium hypochlorite solution per 50 ml sample volume (NaClO, 6–14% active chlorine, Merck Emplura). The samples were then incubated for 24 h at room temperature to remove the residual organic matter that was not digested by hydrogen peroxide. In the following, the sample was relocated to a stainless-steel vacuum filtration system via rinsing with little ultrapure water (Satorius Stedim, 500 ml funnel capacity). After filtration, filters were placed into glass petri dishes and covered with a watch glass until further analysis. |
| --- |


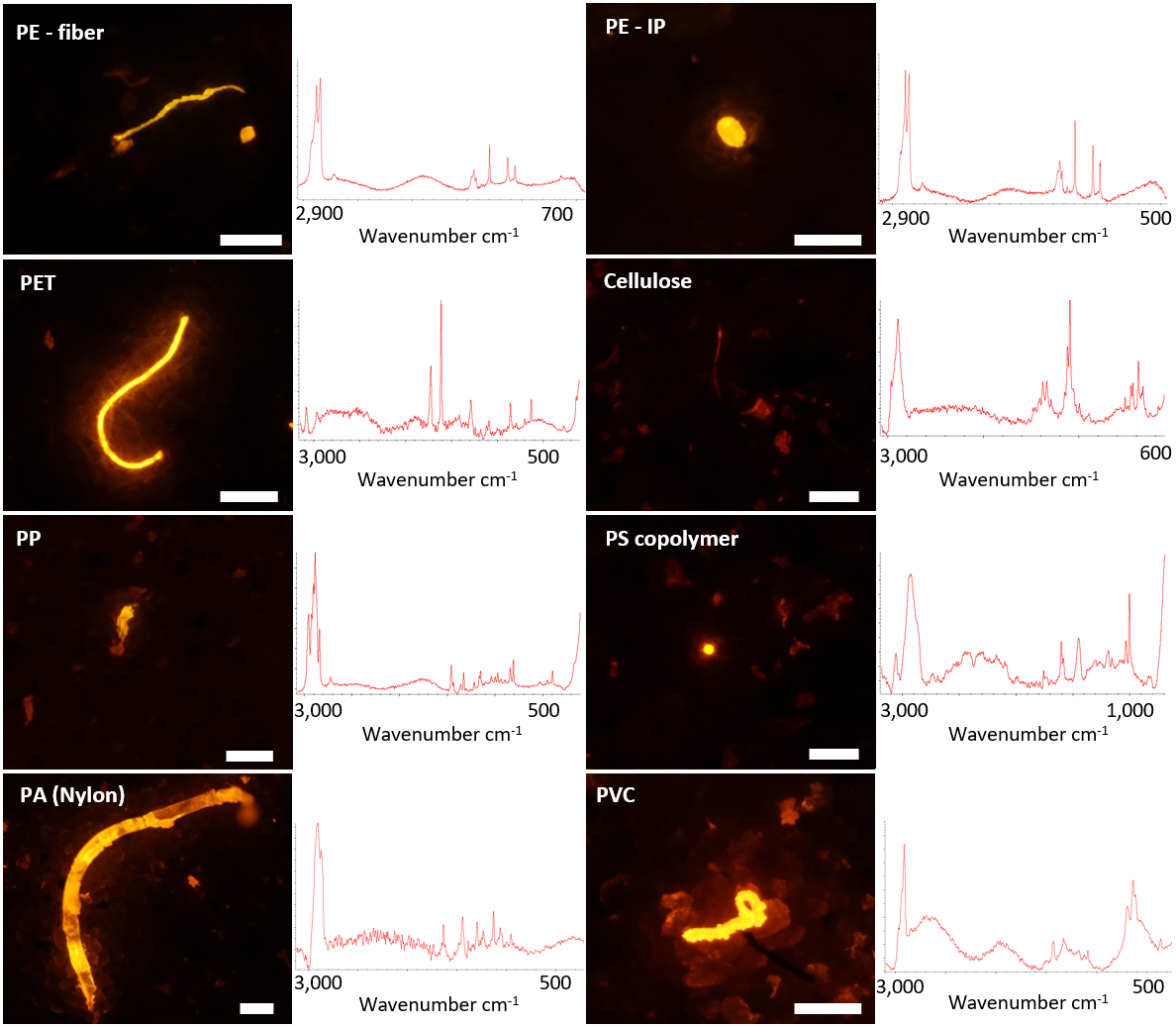


**SI 6** Stained microplastic particles, a cellulose fiber, and associated Raman spectra, white scale bars indicate 200 µm

|  |  |  | Abundance (number) per size class (µm) | | | |
| --- | --- | --- | --- | --- | --- | --- |
| **Campaign** | **Shape** | **Value** | **>63-200** | **>200-300** | **>300-1000** | **>1000-5000** |
| Mar. 2018 (n=3) | IP | Mean | 0.0 | 0.0 | 0.0 | 0.0 |
|  |  | SD | 0.0 | 0.0 | 0.0 | 0.0 |
|  | Fiber | Mean | 0.7 | 0.3 | 0.3 | 0.0 |
|  |  | SD | 0.6 | 0.6 | 0.6 | 0.0 |
| Mar. 2019 (n=10) | IP | Mean | 1.5 | 0.5 | 0.1 | 0.0 |
|  |  | SD | 2.6 | 0.5 | 0.3 | 0.0 |
|  | Fiber | Mean | 0.0 | 0.1 | 0.4 | 0.1 |
|  |  | SD | 0.0 | 0.3 | 0.5 | 0.3 |
| Mar. 2020 (n=5) | IP | Mean | 1.2 | 0.4 | 0.0 | 0.0 |
|  |  | SD | 2.2 | 0.5 | 0.0 | 0.0 |
|  | Fiber | Mean | 0.0 | 0.0 | 1.6 | 0.8 |
|  |  | SD | 0.0 | 0.0 | 1.5 | 0.4 |
|  |  |  | **>20-50** | **>50-300** | **>300-1000** | **>1000-5000** |
| Sep. 2019* (n=20) | IP | Mean | 2.0 | 8.3 | 0.3 | 0.0 |
|  |  | SD | 2.8 | 6.7 | 0.6 | 0.0 |
|  | Fiber | Mean | 0.0 | 1.0 | 0.6 | 0.5 |
|  |  | SD | 0.0 | 1.2 | 1.0 | 0.8 |

**SI 7** Number of microplastics detected in procedural laboratory blanks by shape, size class and sampling campaign, *20 µm-system

|  | **Discharge (m³/s)** | | | | |
| --- | --- | --- | --- | --- | --- |
| **Sampling station** | **Mar. 2018** | **Mar. 2019** | **Sep. 2019** | **Mar. 2020** |  |
| Gaetenbach | 0.907 | 0.27 | 0.084 | 0.647 |  |
| Nonnenbach | 0.633 | 0.116 | 0.013 | 0.313 |  |
| Wustrower Bach | 0.069 | - | - | - |  |
| Tollense NB | 3.38 | 1.58 | 0.711 | 3.282 |  |
| Tollense WO | - | 2.583 | 0.936 | 6.509 |  |
| **Mean** | **1.25** | **1.14** | **0.44** | **2.69** |  |
| **SD** | **1.46** | **1.17** | **0.46** | **2.87** |  |

**SI 8** Discharge measured in the field using the ADC device by sampling station and sampling campaign


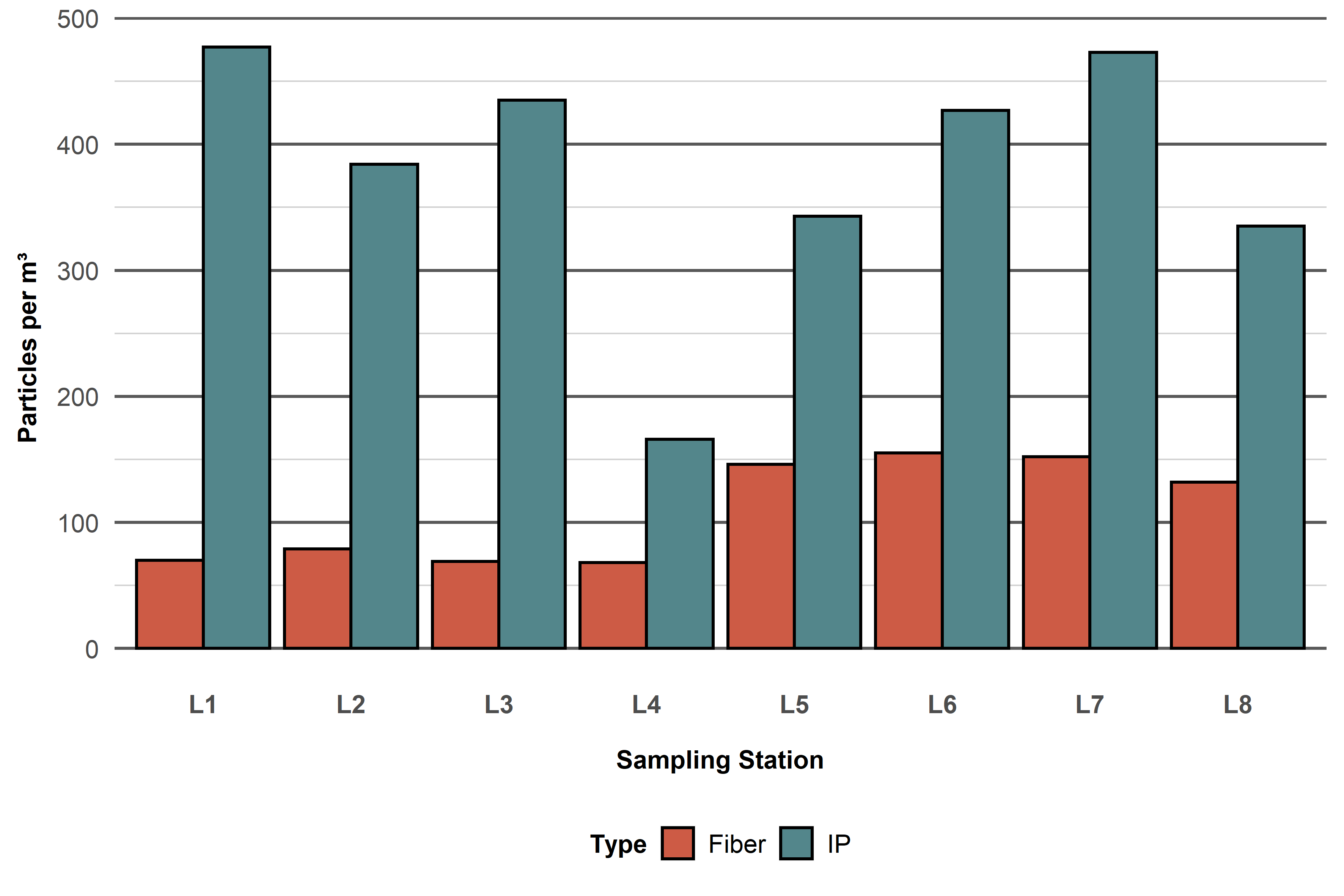


**SI 9** Microplastic concentrations within surface water of Lake Tollense in Sep. 2019 by sampling station and particle shape


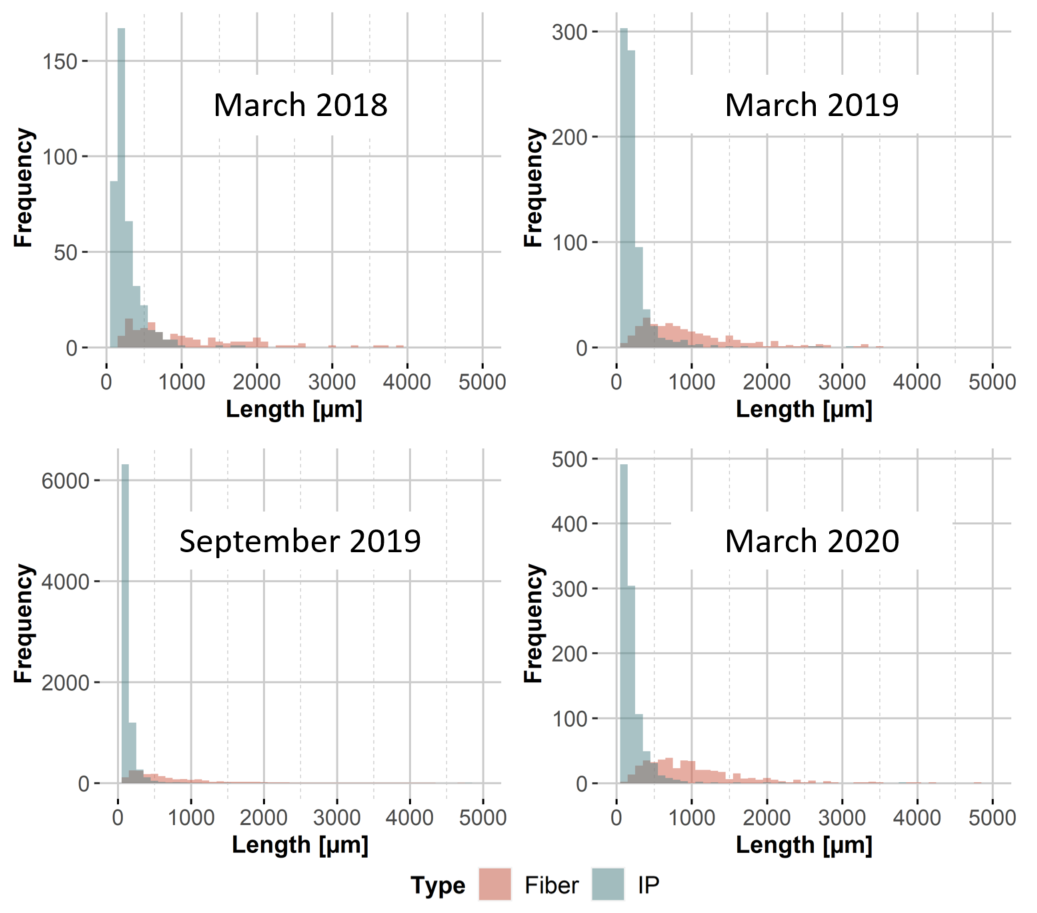


**SI 10** Particle size distributions by sampling campaign and particle shape


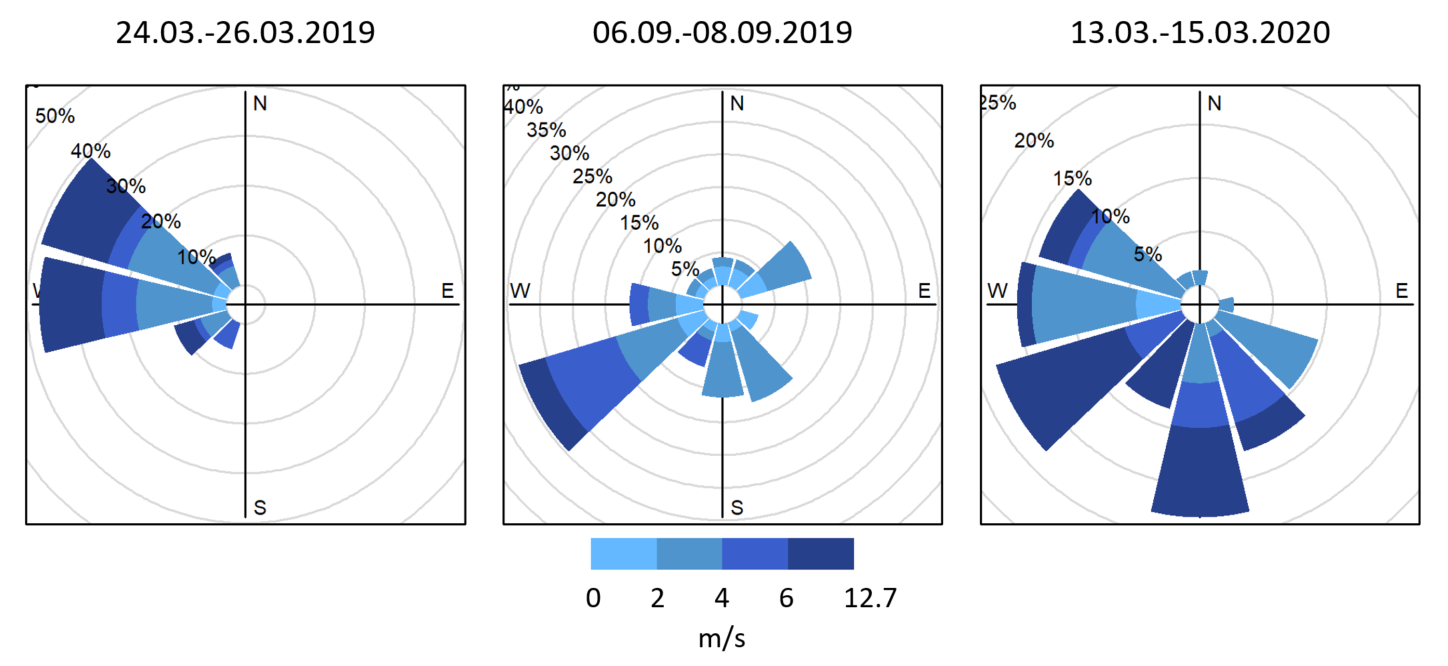


**SI 11** Wind speed and direction at Trollenhagen airport averaged over sampling (at Gaetenbach) days and the two days before (DWD 2021)

**Supporting Information References**

Deutscher Wetterdienst (DWD), 2021. Wind Speed and Wind Direction for Climate Station 5109. Trollenhagen.

European Union, Copernicus Land Monitoring Service, European Environment Agency (EEA) 2021, f.ex. in 2018: “© European Union, Copernicus Land Monitoring Service 2018, European Environment Agency (EEA)"

Hengstmann, E., Tamminga, M., vom Bruch, C., Fischer, E.K., 2018. Microplastic in beach sediments of the Isle of Rügen (Baltic Sea) - Implementing a novel glass elutriation column. Marine Pollution Bulletin 126, 263–274. <https://doi.org/10.1016/j.marpolbul.2017.11.010>

Landesamt für Umwelt, Naturschutz und Geologie Mecklenburg-Vorpommern (LUNG) 2021: Gewässer des digitalen Gewässernetzes M-V (DLM25W). <https://www.umweltkarten.mv-regierung.de/atlas/script/index.php>

Tamminga, M., Hengstmann, E., Fischer, E.K., 2018. Microplastic analysis in the South Funen Archipelago, Baltic Sea, implementing manta trawling and bulk sampling. Marine Pollution Bulletin 128, 601–608. <https://doi.org/10.1016/j.marpolbul.2018.01.066>

Tamminga, M., Stoewer, S.-C., Fischer, E.K., 2019. On the representativeness of pump water samples versus manta sampling in microplastic analysis. Environmental Pollution 254, 112970. <https://doi.org/10.1016/j.envpol.2019.112970>
